# Novel mutations in NSP-1 and PLPro of SARS-CoV-2 NIB-1 genome mount for effective therapeutics

**DOI:** 10.1186/s43141-021-00152-z

**Published:** 2021-04-02

**Authors:** Mohammad Uzzal Hossain, Arittra Bhattacharjee, Md. Tabassum Hossain Emon, Zeshan Mahmud Chowdhury, Ishtiaque Ahammad, Md. Golam Mosaib, Md. Moniruzzaman, Md. Hadisur Rahman, Md. Nazrul Islam, Irfan Ahmed, Md. Ruhul Amin, Asif Rashed, Keshob Chandra Das, Chaman Ara Keya, Md. Salimullah

**Affiliations:** 1 Bioinformatics Division, National Institute of Biotechnology, Ganakbari, Ashulia, Savar, Dhaka, 1349 Bangladesh; 2 Department of Biochemistry and Microbiology, North South University, Bashundhara, Dhaka, 1229 Bangladesh; 3 Department of Biotechnology and Genetic Engineering, Life Science Faculty, Mawlana Bhashani Science and Technology University, Santosh, Tangail, 1902 Bangladesh; 4 Department of Biochemistry and Molecular Biology, Faculty of Health & Medical Sciences, Gono Bishwabidyaloy, Ashulia, Savar, Dhaka, 1344 Bangladesh; 5 Molecular Biotechnology Division, National Institute of Biotechnology, Ganakbari, Ashulia, Savar, Dhaka, 1349 Bangladesh; 6 Plant Biotechnology Division, National Institute of Biotechnology, Ganakbari, Ashulia, Savar, Dhaka, 1349 Bangladesh; 7 Center for Medical Biotechnology, MIS, Directorate General of Health Services, Dhaka, Bangladesh; 8 Department of Microbiology, Mugda Medical College, Dhaka, Bangladesh

**Keywords:** SARS-CoV-2, COVID-19, NSP-1, Papain-like protease, GRL0617, ISG-15

## Abstract

**Background:**

Severe acute respiratory syndrome coronavirus-2 (SARS-CoV-2), the etiologic agent of coronavirus disease 2019 (COVID-19), is rapidly acquiring new mutations. Analysis of these mutations is necessary for gaining knowledge regarding different aspects of therapeutic development. Previously, we have reported a Sanger method-based genome sequence of a viral isolate named SARS-CoV-2 NIB-1, circulating in Bangladesh. The genome has four novel non-synonymous mutations in V121D, V843F, A889V, and G1691C positions.

**Results:**

Using different computational tools, we have found V121D substitution has the potential to destabilize the non-structural protein-1 (NSP-1). NSP-1 inactivates the type-1 interferon-induced antiviral system. Hence, this mutant could be a basis of attenuated vaccines against SARS-CoV-2. V843F, A889V, and G1691C are all located in nonstructural protein-3 (NSP-3). G1691C can decrease the flexibility of the protein. V843F and A889V might change the binding pattern and efficacy of SARS-CoV-2 papain-like protease (PLPro) inhibitor GRL0617. V843F substitution in PLPro was the most prevalent mutation in the clinical samples. This mutation showed a reduced affinity for interferon-stimulated gene-15 protein (ISG-15) and might have an impact on innate immunity and viral spread. However, V843F+A889V double mutant exhibited the same binding affinity as wild type PLPro. A possible reason behind this phenomenon can be that V843F is a conserved residue of PLPro which damaged the protease structure, but A889V, a less conserved residue, presumably neutralized that damage.

**Conclusions:**

Mutants of NSP-1 could provide attenuated vaccines against coronavirus. Also, these mutations of PLPro might be targeted to develop better anti-SARS therapeutics. We hope our study will help to get better insides during the development of attenuated vaccine and PLPro inhibitors.

**Supplementary Information:**

The online version contains supplementary material available at 10.1186/s43141-021-00152-z.

## Background

Severe acute respiratory syndrome coronavirus-2 (SARS-CoV-2), a positive (+) stranded RNA virus that initiated coronavirus disease 2019 (COVID-19) pandemic, is rapidly changing and adapting in different environments [1]. At the beginning of the COVID-19 outbreak, this viral infection was clinically manifested as flu-like symptoms (e.g., cough, fever, and trouble breathing) [2]. However, the acellular pathogen is frequently evolving with novel mutations, and in consequence, viral diarrhea, cardiovascular injury, strokes, psychosis, dementia or Kawasaki like syndrome are becoming more regular in COVID-19 cases [3–5]. According to the World Health Organization (WHO), this constant evolution is taking place due to different selection pressures that are letting the virus to be airborne and more resilient [6]. Proper analysis of these genomic mutations can help to decipher issues regarding viral transmission, epidemiology, pathogenicity, and therapeutics.

Coronaviruses (CoVs) are enveloped viruses that belong to the *Coronaviridae* family and *Orthocoronavirinae* subfamily [7, 8]. Previously reported SARS-CoV-1 and Middle East respiratory syndrome coronavirus (MERS-CoV) belong to the *Betacoronavirus* genus of *Orthocoronavirinae* subfamily [7, 8]. These pathogens were the causative agents of SARS and MERS epidemics. SARS-CoV-2, a member of *Betacoronavirus,* shares nearly 82.30% sequence identity with SARS-CoV-1 while showing strikingly less similarity with MERS-CoV (28%) [9].

The 29.9-kb RNA genome of SARS-CoV-2 has 11 genes with two untranslated regions (UTRs) at 5' and 3' terminals. Nearly 67% of the genome consists of Orf1ab polyprotein gene. The rest of the genome encodes structural proteins (e.g., surface (S), envelope (E), membrane (M), and nucleocapsid (N) proteins) [10]. According to the Global initiative on sharing all influenza data (GISAID) database, with particular prevalent mutations, the virus has made several distinct clades such as S, L, V, G, GR, GV, GRY and GH [11]. Among them, GR is one of the largest and predominates in South America, Europe, and Asia (http://www.gsaid.org). The members of this clade contain S-D614G and N-G204R mutations. Currently, GR is relatively less prevalent in North America. However, we have sequenced a GR-type viral isolate SARS-CoV-2/human/BGD/NIB_01/2020 (SARS-CoV-2 NIB-1) (NCBI GenBank Accession: MT509958, GISAID Accession ID: EPI_ISL_447904) from a young female COVID-19 patient from Bangladesh [12]. According to our report, the viral isolate shares a common ancestor with three North American SARS-CoV-2 isolates that were collected from Washington, United States of America (USA). Additionally, our isolate showed longer branch length or more genomic mutations compared with its closely related members [12]. When we aligned the genome with the reference genome (Accession: NC_045512.2), we found 11 mutations of which 4 of them were new. Amongst the new four mutations, three were found inside nonstructural protein-3 (NSP-3), of which two were located in the papain-like protease (PLPro) domain, and one was situated in the C-terminal domain of NSP-3. The remaining one was detected in nonstructural protein-1 (NSP-1) (Table 1).

**Table 1:** Mutations in SARS-CoV-2 NIB-1

SL no.	Reference position	Position in NIB-1	Reference → NIB-1	Change of amino acid	Coding region/ Gene
**Previously reported mutations**					
1	241	93	**C →** T	-	5' UTR
2	1163	1015	**A →** T	I120F	Orf1ab
3	629	481	**C →** A	L122I	Orf1ab
4	8171	8023	**G →** A	A1818T	Orf1ab
5	23,403	23,255	**A →** G	D614G*	S
6	28,882	28,734	**G →** A	R203K	N
7	28,883	28,735	**G →** C	G204R*	N
**Novel mutations**					
8	627	479	**T →** A	V121D	Orf1ab
9	5246	5098	**G →** T	V843F	Orf1ab
10	5385	5237	**C →** T	A889V	Orf1ab
11	7790	5642	**G →** T	G1691C	Orf1ab

Asterisk (*) containing D614G and G204R made the strain a member of the GR clade.

NSP-1 and PLPro from SARS-CoV-1 or SARS-CoV-2 impede the type 1 interferon (IFN) antiviral activities of the host cells [13]. PLPro cleaves off interferon-stimulating gene-15 (ISG-15) protein; therefore, the host cells cannot execute antiviral signals properly [14, 15]. In this study, we have analyzed the effect of these novel mutations on the respective protein structures. We have shown that some of these mutations have the potential to destabilize the structure of NSP-1 and the C-terminal domain of NSP-3. We also explored how the loss and gain of Valine in V843F and A889V mutations respectively affect the binding of protease inhibitors GRL0617 and ISG-15 for their implication in new therapeutics development.

## Methods

### Genome retrieval and identification of the mutations

The genome of SARS-CoV-2 NIB-1 was collected from the National Center for Biotechnology Information (NCBI) (GenBank accession: MT509958.1). The genome was aligned with the reference genome by NCBI Nucleotide Basic Local Alignment Search Tools (BLASTN) to identify the mutations in the Untranslated Regions (UTRs) [16]. The non-synonymous mutations were collected from GISAID CoVsurver (http://www.gisaid.org).

### Effect of the mutations on the proteins

Mutations that affect the structural stability of the proteins can behave differently than the wild type one [17, 18]. To assess the effect of the novel mutations, MUpro, Protein Variation Effect Analyzer (PROVEAN), and HOPE were employed [19–21]. MUpro implemented Support Vector Machine and Neural Network to predict the results of the mutations. HOPE and PROVEAN used different programs to evaluate the possible outcomes due to the change of the amino acids in the proteins. Later, PLPro was taken for further analysis because of the availability of structural and molecular mechanisms on this protein.

### *In silico* mutagenesis, molecular modeling, and refinement of PLPro

To observe the mutational effect of the protein at a three-dimensional (3D) level, the crystal structure of PLPro was collected from Research Collaboratory for Structural Bioinformatics (RCSB) Protein Data Bank (PDB) (http://www.rcbs.org) [22]. The chain A of PLPro (PDB ID: 6W9C) was preserved via BIOVIA Discovery Studio (https://www.3ds.com/). Heterogeneous atoms were also removed. Mutated protein sequences went under homology modeling by SWISS-MODEL using chain A of 6W9C as a template [23]. The generated structures were energetically minimized by 3D Refine and Galaxy Refine [24, 25].

### Structural assessment of mutant proteins

The structural quality of the mutant proteins was evaluated. To execute this step, RAMPAGE, ERRAT, VERIFY3D, PROVE, and SWISS-MODEL structure assessments were employed [23, 26–29].

### Inhibitor binding analysis against the wild type and mutant PLPros

GRL0617 or 5-Amino-2-methyl-N-[(R)-1-(1-naphthyl) ethyl] benzamide (PubChem CID: 24941262) is a PLPro inhibitor with a relatively low level of cytotoxicity [30]. This naphthalene-based small molecule has the potential to be a prospective antiviral drug. Here, we analyzed the interactions between GRL0617 and PLPro by AutoDock tools 1.5.6 using our previously reported methodology for exploring drug–receptor interactions [31, 32]. In short, the GRL0616 was taken as a ligand, while the wild type and mutant proteins were used as receptors. The active site of the palm domain in PLPro was enclosed with Grid Box [33]. The wild type PLPro (dimension points: *X* = 92, *Y* = 74, *Z* = 112) was enclosed with 0.425 Å spacing. A889V (dimension points: *X* = 60, *Y* = 74, *Z* = 96) and V843F mutants (dimensions points: *X* = 64, *Y* = 86, *Z* = 102) were enclosed with 0.531 and 0.499 Å spacing, respectively. V843+A889V double mutant (dimension points: *X* = 92, *Y* = 88, *Z* = 112) had 0.375 Å spacing (detail parameters are given in Supplementary file 1). The molecular interactions between GRL0617-PLPro were visualized by UCSF Chimera [34], BIOVIA Discovery Studio and PyMOL Molecular Graphics System, Version 2.3.3 Schrödinger, LLC. Molecular dynamics (MD) simulations were carried out using Newtonian equations of motion via GROMACS 2020.1 [35]. Here, the PDB files of drug–receptor complexes were energetically minimized using GROMOS96 43a1 force field [36]. The protein topologies were built using pdb2gmx module within GROMACS, while the ligand topology was built using the PRODRG 2.5 server [37]. The protein–ligand complexes were solved in simple point charge (SPC) water model within a dodecahedron [38]. A minimum of 1 nanometer (nm) distance was maintained between the protein surfaces and the edges of the box inside the unit cells. Each system was neutralized by adding 3 chlorine ions. Then the energy minimization was performed using the “grompp” module. The maximum force allowed was kept below 10 kJ/mol/nm tp. After restraining the ligand and setting up temperature-coupling groups by combining the protein and the inhibitor, isothermal–isochoric (NVT) equilibration were done for 100 picoseconds (ps). Following the NVT equilibration, isothermal–isobaric (NPT) equilibration were carried out for the same duration. Finally, 50 nanoseconds (ns) of molecular dynamic simulation was carried out using 300 K temperature, 1 bar pressure, 1.2, short-range Van der Waals cutoff of 1.2 nm, Particle Mesh Ewald for long-range electrostatics, and periodic boundary conditions. The trajectories of atoms were recorded every 2 femtoseconds (fs).

### Analysis of ISG-15-PLPro interactions

ISG-15 C-terminal domain that interacts with PLPro was collected from PDB ID: 6XA9. As a ligand/substrate, this protein was docked against the PLPro receptors by GalaxyTongDock_A [39]. After docking, Model 1 PDB files were taken since they had the highest cluster sizes and docking scores. The selected models were further analyzed by PROtein binDIng enerGY prediction (PRODIGY) to determine the binding affinity of the protein complexes at 25 °C temperature [40].

### Detection of the novel mutations in clinical samples

To identify the presence of these mutations in other SARS-CoV-2 isolates, we have collected 27 samples from July 2020 to November 2020 with their clinical data. All of the patients were tested positive for COVID-19 via Reverse transcription-polymerase chain reaction (RT-PCR). The SARS-CoV-2 viral RNA was extracted from patient’s specimen using the PureLink viral RNA/DNA minikit (Invitrogen). After that, the RNA was converted into cDNA by SuperScript VILO cDNA synthesis kit (Invitrogen). The specific regions that had novel mutations were gone under PCR amplification using our previously reported primers [12]. The amplicons were visualized via 1.5% agarose gel electrophoresis and purified using the PureLink PCR purification kit (Thermo Fisher Scientific, USA). Sanger dideoxy method was implemented to sequence the purified amplicons with 2× coverage. ABI 3500 and BigDye Terminator version 3.1 cycle sequencing kit (Applied Biosystems, USA) were used for sequencing.

## Results

**Fig. 1:**
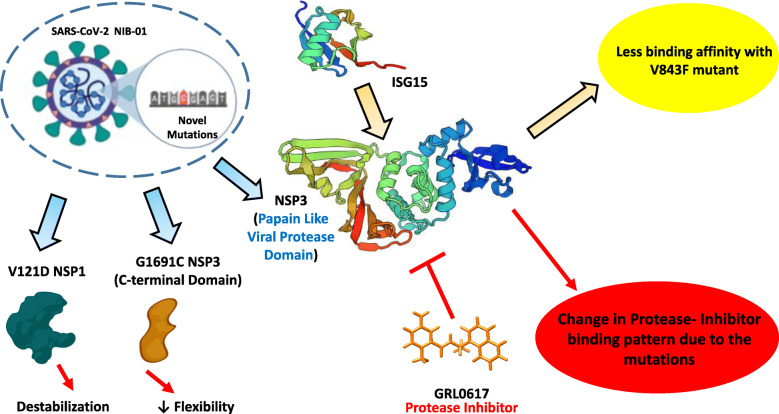
Graphical representation of the whole work. Some of the parts of this figure were generated with the help of BioRender (https://biorender.com/)

The overall scheme of the present study has been described in Fig. 1.

### Transversion mutations are predominant in SARS-CoV-2 NIB-1

SARS-CoV-2 NIB-1 is a member of the GR clade (Table 1). The genome contains 11 mutations in total. Among them, 6 were transversion mutations, and 5 were transition mutations. According to GISAID, 7 of these mutations were reported previously, and 4 of them were first identified in SARS-CoV-2 NIB-1 isolate. In these 7 mutations, 4 were transition mutations: three Purine → Purine transitions (Table 1; Serial # 4–6) and one Pyrimidine → Pyrimidine transition (Table 1; Serial # 1). The other three mutations were transversion mutations: two Purine → Pyrimidine transversions (Table 1; Serial # 2, 3) and one Pyrimidine → Purine transversion (Table 1; Serial # 7). The 4 novel mutations were located in the Orf1b gene. Among them, only A889V substitution occurred due to C → T (Pyrimidine → Pyrimidine) transition. The other 3 mutations were transversion substitutions: two Purine → Pyrimidine transversions (Table 1; Serial # 9, 11) and one Pyrimidine → Purine transversion.

### Novel mutations have the potential to alter the functions and structures of protein

According to the MUpro, V121D, V843F, and G1691C can destabilize the proteins (Table 2). Moreover, PROVEAN determined that the V121D and G1691C substitutions are deleterious for the biological functions of the proteins. HOPE demonstrated that most of the mutant amino acids are located in a conserved region, and they are bigger than the wild-type residues. They have the potential to abolish the protein functions. In contrast, only A889V mutant PLPro can increase protein stability (Table 2).

**Table 2:** Effect of the novel mutations in protein stability, function, and structure

SL no.	Change of amino acid	Protein stability (MUpro)	Protein function (PROVEAN; cutoff = − 2.5)	Protein structural properties (HOPE)
1	V121D	Decrease	Deleterious	**Change of amino acid:** Neutral **→** Negative **Change in structural stability:** Slight destabilization of NSP-1 **Level of conservation:** Highly conserved
2	V843F	Decrease	Neutral	**Change of amino acid size:** Mutant residue size is bigger **Change in structural stability:** Disturbing the domain and might abolish its function **Level of conservation:** Highly conserved
3	A889V	Increase	Neutral	**Change of amino acid size:** Mutant residue size is bigger. **Change in structural stability:** Disturbing the domain and might abolish its function **Level of conservation:** Located near a highly conserved position, this mutation might occur without damaging the protein.
4	G1691C	Decrease	Deleterious	**Change of amino acid size:** Mutant residue size is bigger and more hydrophobic. **Change in structural stability**: The wild-type residue is a glycine that might be necessary to give the protein essential flexibility. Mutation of this glycine might abolish the function of the protein. **Level of conservation**: The mutant residue is located near a highly conserved position. The mutant and wild-type residue are not very similar. Based on this conservation information, this mutation is probably damaging to the protein.

### Interaction patterns of GRL0617-PLPro altered in mutant proteases

**Fig. 2:**
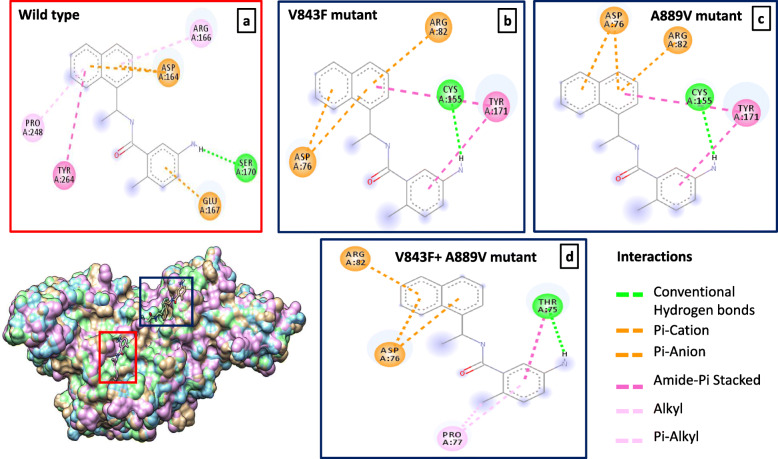
Interactions between GRL0617 inhibitor and superimposed SARS-CoV-2 wild type and mutant papain-like viral proteases (**a–d**). Interacting amino acid residues in wild-type and PLPro mutant SARS-CoV-2. Inhibitor binding into the wild type and mutant protein. The red and blue boxes indicate the location and the interactions between ligand and receptors in wild and in mutant proteases, respectively

The interactions between the inhibitor GRL0617 and PLPro were identified via molecular docking simulation (Fig. 2). Before molecular docking, the quality of the receptors was evaluated by analyzing Ramachandran plots, ERRAT, PROVE, and MolProbity scores. Ramachandran plots showed that the mutant PLPros have 98.1–98.4% amino acids in favored regions, 1.6–1.9% in allowed regions, and 0% in outlier regions. Moreover, the overall quality score of ERRAT was 89.160–91.034% and mean Z score was 0.45–0.50. All of the mutants passed the VERIFY3D evaluations. The MolProbity score of the wild-type crystal structure was 2.55, while the mutants have MolProbity scores between 1.16 and 1.32 (Supplementary file 3). Therefore, after the homology modeling, the quality of the simulated structures was satisfactory for further analysis [26, 41].

V843F PLPro (− 6.7 kcal/mol) and A889V PLPro (− 6.9 kcal/mol) showed more binding affinity scores than the wild type one (− 6.6 kcal/mol) against GRL0617 (Table 3). However, V843F + A889V PLPro showed slightly less affinity (− 6.2 kcal/mol) than others.

**Table 3:** Interaction between wild-type and mutant SARS-CoV-2 papain-like viral protease with GRL0617

Receptor	Binding affinity score (kcal/mol)	Participating amino acids in interaction
Wild type	− 6.6	ASP164, ARG166, GLU167, *SER170*, PRO248, TYR264
V843F	− 6.7	ASP76, ARG82, *CYS155*, TYR171
A889V	− 6.9	ASP76, ARG82, *CYS155,* TYR171
V843F + A889V	− 6.2	ASP76, ARG82, *THR75*, PRO77

*Italicized residues interacted via hydrogen bonds.*

Although the binding affinity did not change significantly due to the mutations, the inhibitor binding site changed along with the interacting amino acids (Table 3). The wild-type receptor interacted with ASP164, ARG166, GLU167, SER170, PRO248, and TYR264, whereas ASP76 and ARG82 were common interacting residues for mutant type receptors. Moreover, residues that participated in hydrogen bonding in wild type, single mutants, and double mutants were not the same. V843F and A889V single mutants have the same interacting residue CYS155 which interacted with the inhibitor through hydrogen bonding.

GRL0617 bound mutant PLPros exhibited nearly same root-mean-square deviations (RMSD) value at 50 ns. However, V843F + A889V double mutant exhibited a slightly higher value at the end. The mutations significantly altered the root-mean-square fluctuations (RMSF) of the amino acid region: 250 to 300. In mutated regions, no remarkable changes of mobility were found. A889V showed a notably altered radius of gyration (Rg) (Supplementary file 5).

### V843F PLPro was the most common mutant that significantly reduced the affinity toward ISG-15

SARS-CoV-2 PLPros cleaves off ISG-15 that is essential to take over the type 1 IFN-mediated antiviral responses [14]. The wild type, A889V and V843F + A889V double mutants showed nearly the same binding affinity (− 15.4 to − 15.5 kcal/mol) toward the C-terminal domain of ISG-15. However, V843F mutant exhibited a significantly lower docking score and binding affinity (− 11.1 kcal/mol) with higher dissociation constant (Table 4). When these mutations were screened in 27 clinical samples, 8 samples (~ 29%) showed V843F mutation in PLPro. The Sanger sequencing reads for both wild type and V843F are given in Supplementary file 4. These V843F-positive patients had mild to moderate symptoms and did not require any intensive care from hospitals.

**Table 4:** Interaction between wild-type and mutant SARS-CoV-2 papain-like viral protease with ISG-15

Receptor	TongDock_A score	Cluster size	Binding affinity score (kcal/mol)	Dissociation constant (K_**d**_) (M)
Wild type	915.278	41	− 15.4	4.8E–12
V843F	720.354	23	− 11.1	7.2E–09
A889V	1019.921	31	− 15.4	4.7E–12
V843F + A889V	968.296	28	− 15.5	4.2E–12

## Discussion

SARS-CoV-2 is a novel coronavirus and mostly resembles SARS-CoV-1 (~80% sequence similarity). In this study, we focused on SARS-CoV-1-related reports to analyze the results of the mutations [9, 42]. Moreover, we also explored other well-studied *betacoronavirus* members such as Middle East respiratory syndrome (MERS-CoV), human coronavirus OC43, and mouse hepatitis coronavirus (MHV) to comprehend different genomic and proteomic functions of SARS-CoV-2.

GISAID CoVsurver revealed that our previously reported isolate ‘SARS-CoV-2 NIB-1’ is a member of GR clade which mutated 10 times in the coding regions [12]. The NCBI BLASTN unveiled another mutation in the 5' UTR region. Generally, species are usually biased to transition mutations but in this viral isolate, 54% of changes occurred due to transversion mutations (Table 1). Transversion mutations are detrimental for RNA viruses (e.g., influenza A and human immunodeficiency viruses (HIV)) since they can radically change the amino acids [43, 44].

The novel mutations V121D, V843F, and G1691C occurred in SARS-CoV-2 NIB-1 isolate due to transversion substitutions that have the potentials to destabilize or alter the protein structure and functions (Table 2). Especially, V121D and V843F positions are highly conserved in the protein, and G1691C might reduce the essential flexibility of NSP-3 (Table 2). Amino acid substitution in V843F and G1691C took place due to G → T transversion that was possibly introduced by Oxo-guanine generated from reactive oxygen species (ROS) [43–45]. Conversely, C → T transition, the most frequent transition of SARS-CoV-2, increased the structural stability of PLPro (Table 2) [45]. Besides, the C **→** T transition in the 5' UTR region might interfere with the function of N and NSP-1 protein [46].

SARS-CoV-2 NIB-1 has V121D and L122I amino acid substitutions in the NSP-1. SARS-CoV-2 NSP-1 has 84.4% similarity with SARS-CoV-1 NSP-1 [47]. NSP-1 is a potent virulence factor of SARS-CoV-1 that reduces the host mRNA expression by binding with the host 40S ribosome to inhibit the translation and specifically accelerate host mRNA degradation keeping the viral mRNA intact [48, 49]. NSP-1 also disrupts the activation of IFN-dependent antiviral signaling pathways and represses the expression of the innate immune-responding genes such as type I IFN, ISG-56, and ISG-15 [13, 50, 51]. These IFN and ISG-15 gene-mediated antiviral pathways are crucial in host defense mechanisms against SARS-CoV-1 and SARS-CoV-2 [15, 50, 52]. An attenuated SARS-CoV-1 with mutant NSP-1 can replicate as efficiently as wild-type strains with an intact IFN response [52]. Moreover, mutant NSP-1 of MHV exhibited an adequate amount of cytotoxic T cell generations that protected the mice against further viral infections [53]. Hence, there is a higher possibility that a suitable NSP-1 mutant of SARS-CoV-2 would work as an attenuated vaccine for COVID-19. Through computational analysis, we have observed that V121D and L122I mutants might destabilize the structure of NSP-1 (Table 2 & Supplementary 2) yet with these two mutations, the virus caused mild fever, cough, and throat congestion in a young female patient [12]. Therefore, V121D and L122I mutations along with 93rd C **→** T in 5' UTR did not change the pathogenicity of SARS-CoV-2 significantly. However, keeping in mind that the quick recovery of the patient within 10 days of the onset of symptoms without any notable care, it might be speculated that NSP-1 with these mutations can be a potent candidate for the investigations and development of an attenuated vaccine against SARS-CoV-2.

**Fig. 3:**
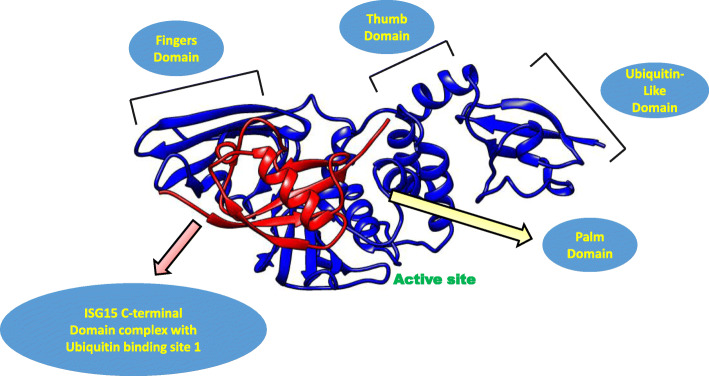
Interactions between the C-terminal domain of interferon-stimulated gene 15 (ISG-15) and SARS-CoV-2 wild-type papain-like viral proteases. The red chain is the C-terminal domain of ISG-15. The blue chain represents the protease

Main protease and PLPro are two essential viral enzymes that are vital for polypeptide processing during viral maturation [47]. Therefore, inhibitors of these proteins could yield prospective antiviral drugs [54]. SARS-CoV-1 PLPro has been targeted to develop inhibitors for decades [33]. SARS-CoV-1 and SARS-CoV-2 PLPros have 4 domains (Fig. 3) [30]. The active site of this enzyme resides in the thumb domain. PLPro interacts with the N- and C-terminal domains of ISG-15 through ubiquitin-binding subsites 2 and 1, respectively [33]. This interaction finally cleaves off ISG-15. Hence, type 1 IFN-induced ISG-15 antiviral response cannot function properly in host cells. Consequently, this process hinders innate immune response and enhances viral spread [14]. A study also demonstrated that SARS-CoV-2-infected Vero and Calu3 2B4 cell lines are more susceptible to type 1 INF treatment than SARS-CoV-1 [50, 51]. Most possibly, the IFN treatment increased the synthesis of ISG-15 that ceased the viral replications in the cell lines. PLPro-mediated pathogenesis can be halted with naphthalene-based protease inhibitor GRL0617 since this ligand can make non-covalent bonds with SARS-CoV-2 PLPro [14, 30]. Here, V843F and A889V mutations were present in the PLPro domain of the NSP-3 protein. V843F and A889V single mutants and V843F + A889V double mutant changed the binding site of GRL0617. The inhibitor binds in the thumb domain of the wild-type receptor, whereas the mutant receptors interacted through the ubiquitin-like domain (Fig. 2). Although GRL0617-bound mutant PLPros showed similar RMSD at 50 ns, V843F + A889V double mutant demonstrated a slightly higher value at the end. The mutations significantly reduced the mobility of the amino acid region: 250 to 300. According to the PDB ID: 6W9C/ Uniprot ID: P0DTD1, this region has an important role in GRL0617-induced inhibition as mutation in 268th position reduced GRL0617 activity. Additionally, 272th H is an active site residue. Therefore, these mutations might decrease the efficacy of GRL0617. A889V can change the compactness of the proteins since it showed a notably altered Rg during the MD simulation [55].There is a chance that these altered binding positions, RMSF, Rg, and RMSD can make the virus resistant against GRL0617. Therefore, GRL0617 may not work well against all variants of SARS-CoV-2.

The mutants of PLPro were also docked against the C-terminal domain of ISG-15. To execute this step, the ISG-15 portion was separated from the PLPro-ISG-15 C-terminal domain complex (PDB ID: 6XA9). Mutant A889V, V843F + A889V, and wild-type PLPro did not show any differences when docked against ISG-15. However, the most frequent mutation V843F led to reduced binding affinity towards the ubiquitin-binding subsite 1 of SARS-CoV-2 PLPro. According to the clinical histories, V843F did not raise pathogenesis in the patients. Therefore, V843F can be assumed as a detrimental and destabilizing transversion mutation that reduced the degradation of ISG-15. Efficient degradation of ISG-15 is a major step to annihilate the innate immune responses. V843F mutation can reduce this process and allow the host cells to halt viral replications. In SARS-CoV-2 NIB-1, A889V substitution protected the protease from the damage of V843F. A889V occurred due to the most common C → T transition; plausibly this mutation first appeared in the protein, and then V843F took place due to ROS. Yet, further studies are needed to explore how this “yin and yang” incident affects the viral physiology. We hope our findings will give better insights during the development of attenuated vaccines and antiviral drugs.

## Conclusion

This study focused on the effects of novel mutations in SARS-CoV-2 NIB-1 genome. The genome has 4 novel mutations in NSP-1 and NSP-3. These proteins perform essential roles to antagonize IFN responses. A defective or weaker version of these proteins will yield clear insights regarding viral pathogenesis, the pattern of transmission, epidemiology, and therapeutics development.

Our novel mutations will provide helpful insights to create weakened versions of different variants that will emerge in upcoming years. Moreover, our analyses will help to design better universal inhibitors, suitable drugs, or mutant-specific antivirals against PLPro.

## Supplementary Information

**Additional file 1: Supplementary File 1.** Parameters for Molecular Docking. The number of Points in Dimension, Spacing (Angstrom) and Center Grid Box values that were taken during the molecular docking of Wild type/ Mutant PLPros and GRL0617.

**Additional file 2: Supplementary File 2.** Effect of the Mutations in NSP1 Protein Stability, Function and Structure. MUpro, PROVEAN and HOPE results for mutant L122I NSP1.

**Additional file 3: Supplementary File 3.** Structural quality reports of Mutant PLPros. The structural quality reports of V843F, A889V and A889V evaluated by RAMPAGE, MolProbity, ERRAT and PROVE.

**Additional file 4: Supplementary File 4.** Sanger Sequencing Reads for Wild Type and Mutant SARS-CoV-2 PLPro. Sequencing Reads of SARS-CoV-2 PLPro V843 coding regions for both Wild type and Mutant isolates. Around 29% samples showed **G →** T transversion or V843F substitution.

**Additional file 5: Supplementary File 5.** Results from Molecular Dynamics (MD) Simulations. RMSD, RMSF and Rg values of GRL0617 bound wild type and mutant PLPro enzymes.

## Data Availability

All of the data are included in the article.

## Abbreviations

SARS-CoV: Severe acute respiratory syndrome coronavirus
PLPro: Papain-like protease
IFN: Interferon
ISG-15: Interferon-stimulating gene-15
PROVEAN: Protein Variation Effect Analyzer
ROS: Reactive oxygen species
PRODIGY: Protein binding energy prediction
