# Supplementary material for: Novel mutations in NSP-1 and PLPro of SARS-CoV-2 NIB-1 genome mount for effective therapeutics
Source: J Genet Eng Biotechnol. 2021 Apr 2;19:52. doi: 10.1186/s43141-021-00152-z (PMC8017899; doi:10.1186/s43141-021-00152-z)
Supplement: Supplementary file 2 — Additional file 2: Supplementary File 2. Effect of the Mutations in NSP1 Protein Stability, Function and Structure. MUpro, PROVEAN and HOPE results for mutant L122I NSP1. [file 43141_2021_152_MOESM2_ESM.docx]

| **SL No.** | **Change of Amino Acid** | **Protein Stability (MUPro)** | **Protein Function (PROVEAN;**  cutoff= -2.5) | **Protein Structural Properties (HOPE)** |
| --- | --- | --- | --- | --- |
| 1 | L122I | DECREASE | Neutral | **Change of Amino Acid Size:**  No Change  **Level of Conservation:**  Highly Conserved |

**Effect of the mutations in NSP1 protein stability, function and structure.**
