# Supplementary material for: Novel mutations in NSP-1 and PLPro of SARS-CoV-2 NIB-1 genome mount for effective therapeutics
Source: J Genet Eng Biotechnol. 2021 Apr 2;19:52. doi: 10.1186/s43141-021-00152-z (PMC8017899; doi:10.1186/s43141-021-00152-z)
Supplement: Supplementary file 3 — Additional file 3: Supplementary File 3. Structural quality reports of Mutant PLPros. The structural quality reports of V843F, A889V and A889V evaluated by RAMPAGE, MolProbity, ERRAT and PROVE. [file 43141_2021_152_MOESM3_ESM.pdf]

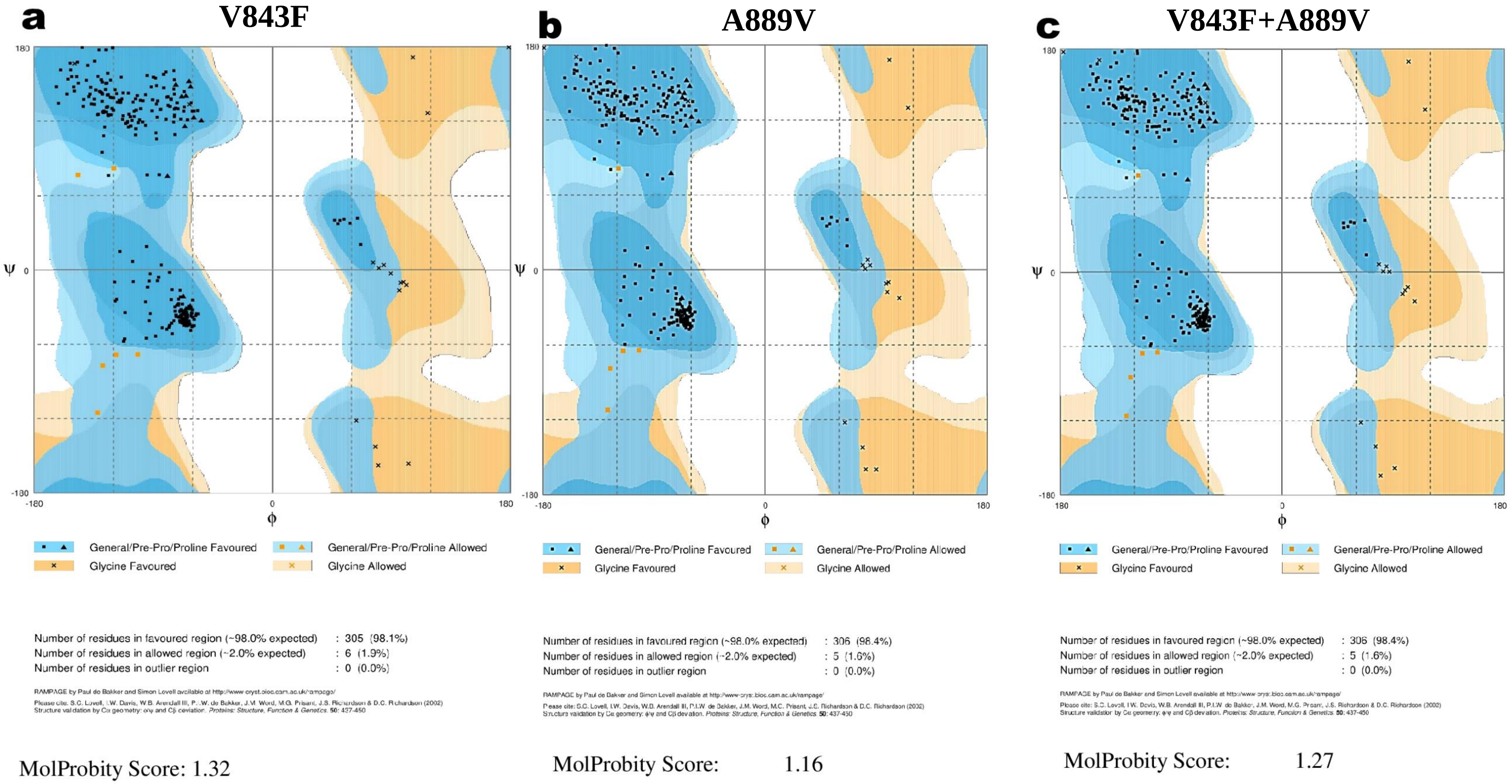
a
V843F
b
A889V
c
V843F+A889V
ψ
φ
180
0
-180
General/Pre-Pro/Proline Favoured
General/Pre-Pro/Proline Allowed
Glycine Favoured
Glycine Allowed
Number of residues in favoured region (~98.0% expected) : 305 (98.1%)
Number of residues in allowed region (~2.0% expected) : 6 (1.9%)
Number of residues in outlier region : 0 (0.0%)
Number of residues in favoured region (~98.0% expected) : 306 (98.4%)
Number of residues in allowed region (~2.0% expected) : 5 (1.6%)
Number of residues in outlier region : 0 (0.0%)
Number of residues in favoured region (~98.0% expected) : 306 (98.4%)
Number of residues in allowed region (~2.0% expected) : 5 (1.6%)
Number of residues in outlier region : 0 (0.0%)
RAMPAGE by Paul de Bakker and Simon Lovell available at http://www-cryst.bioc.cam.ac.uk/rampage/
Please cite: S.C. Lovell, I.W. Davis, W.B. Arendall III, P.I.W. de Bakker, J.M. Word, M.G. Prisant, J.S. Richardson & D.C. Richardson (2002)
Structure validation by Cα geometry: φ/ψ and Cβ deviation. Proteins: Structure, Function & Genetics. 50: 437-450
MolProbity Score: 1.32
MolProbity Score: 1.16
MolProbity Score: 1.27

V843F

ERRAT

Overall Quality Factor

91.0345

VERIFY3D

98.72% of the residues have averaged 3D-1D score >= 0.2

Pass

Z–score mean	0.452
Z–score stddev	1.189
Z–score RMS	1.271
# scored atoms	1174
# outliers	36
% outliers	3.100

A889V

ERRAT

Overall Quality Factor

89.3103

VERIFY3D

96.49% of the residues have averaged 3D-1D score >= 0.2

Pass

Z–score mean	0.454
Z–score stddev	1.218
Z–score RMS	1.299
# scored atoms	1194
# outliers	43
% outliers	3.600

V843F+A889V

ERRAT

Overall Quality Factor

89.1608

VERIFY3D

94.25% of the residues have averaged 3D-1D score >= 0.2

Pass

Z–score mean	0.503
Z–score stddev	1.268
Z–score RMS	1.363
# scored atoms	1211
# outliers	55
% outliers	4.500
