# Supplementary material for: Novel mutations in NSP-1 and PLPro of SARS-CoV-2 NIB-1 genome mount for effective therapeutics
Source: J Genet Eng Biotechnol. 2021 Apr 2;19:52. doi: 10.1186/s43141-021-00152-z (PMC8017899; doi:10.1186/s43141-021-00152-z)
Supplement: Supplementary file 4 — Additional file 4: Supplementary File 4. Sanger Sequencing Reads for Wild Type and Mutant SARS-CoV-2 PLPro. Sequencing Reads of SARS-CoV-2 PLPro V843 coding regions for both Wild type and Mutant isolates. Around 29% samples showed G → T transversion or V843F substitution. [file 43141_2021_152_MOESM4_ESM.pdf]

Wild Type PLPro (n=19) **V843F Mutant PLPro (n=8)**

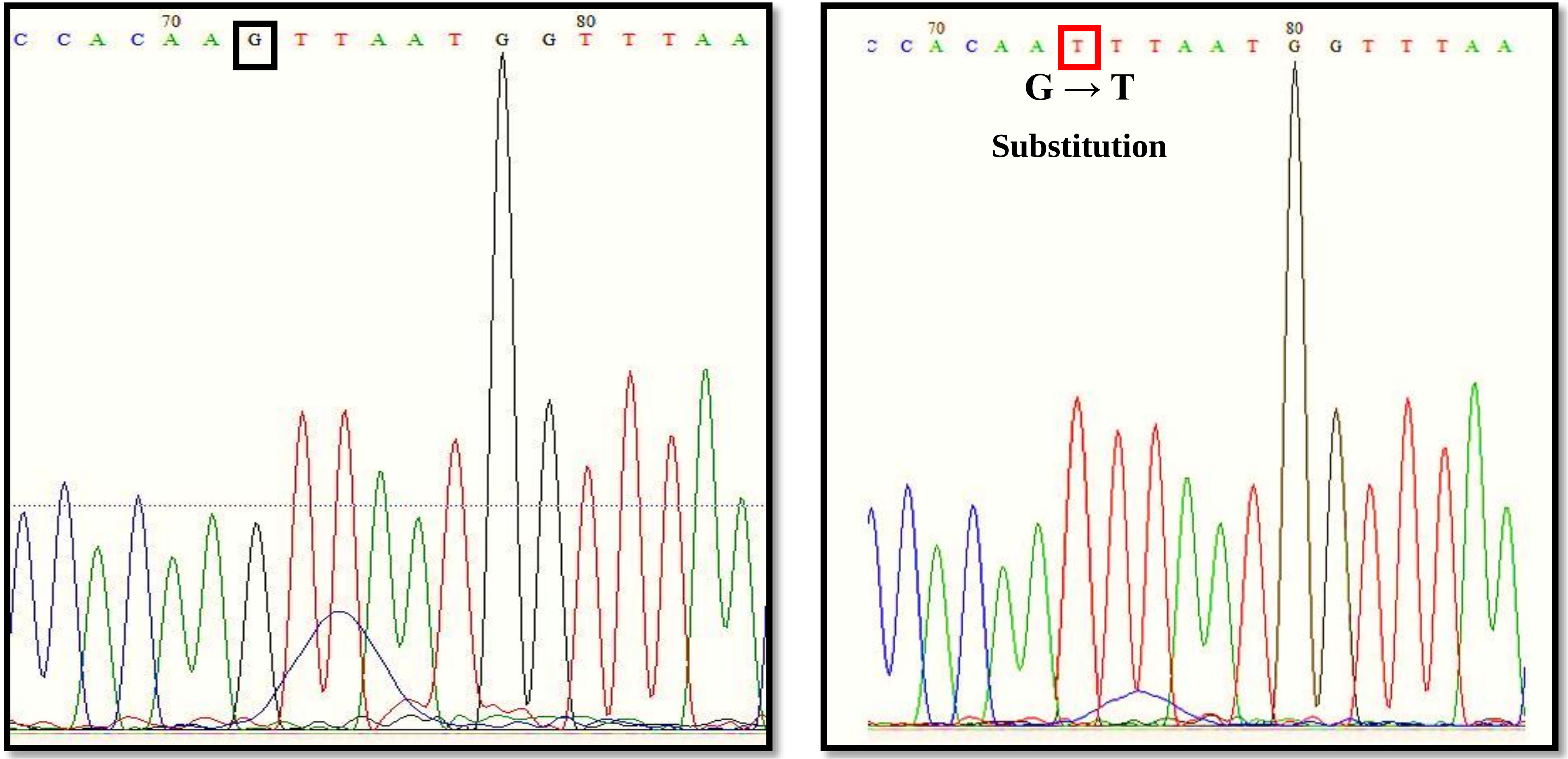

Sanger Sequencing reads of SARS-CoV-2 PLPro V843 coding regions
for both Wild type and Mutant isolates. Here “ n” represents the number of patients.
