# Supplementary material for: Novel mutations in NSP-1 and PLPro of SARS-CoV-2 NIB-1 genome mount for effective therapeutics
Source: J Genet Eng Biotechnol. 2021 Apr 2;19:52. doi: 10.1186/s43141-021-00152-z (PMC8017899; doi:10.1186/s43141-021-00152-z)

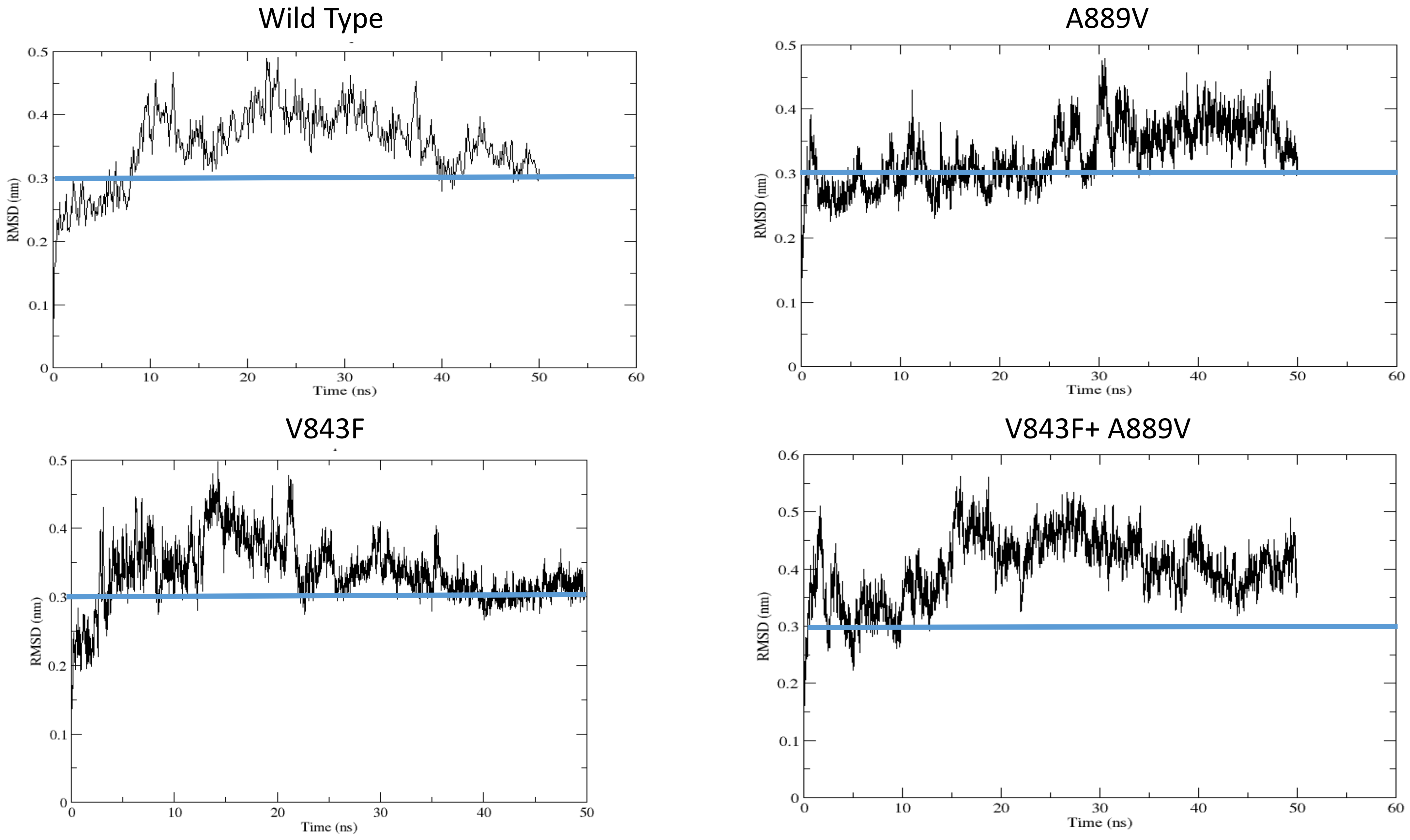

Root-Mean-Square Deviation (RMSD) of the GRL0617 docked with wild type and Mutant PLPros.

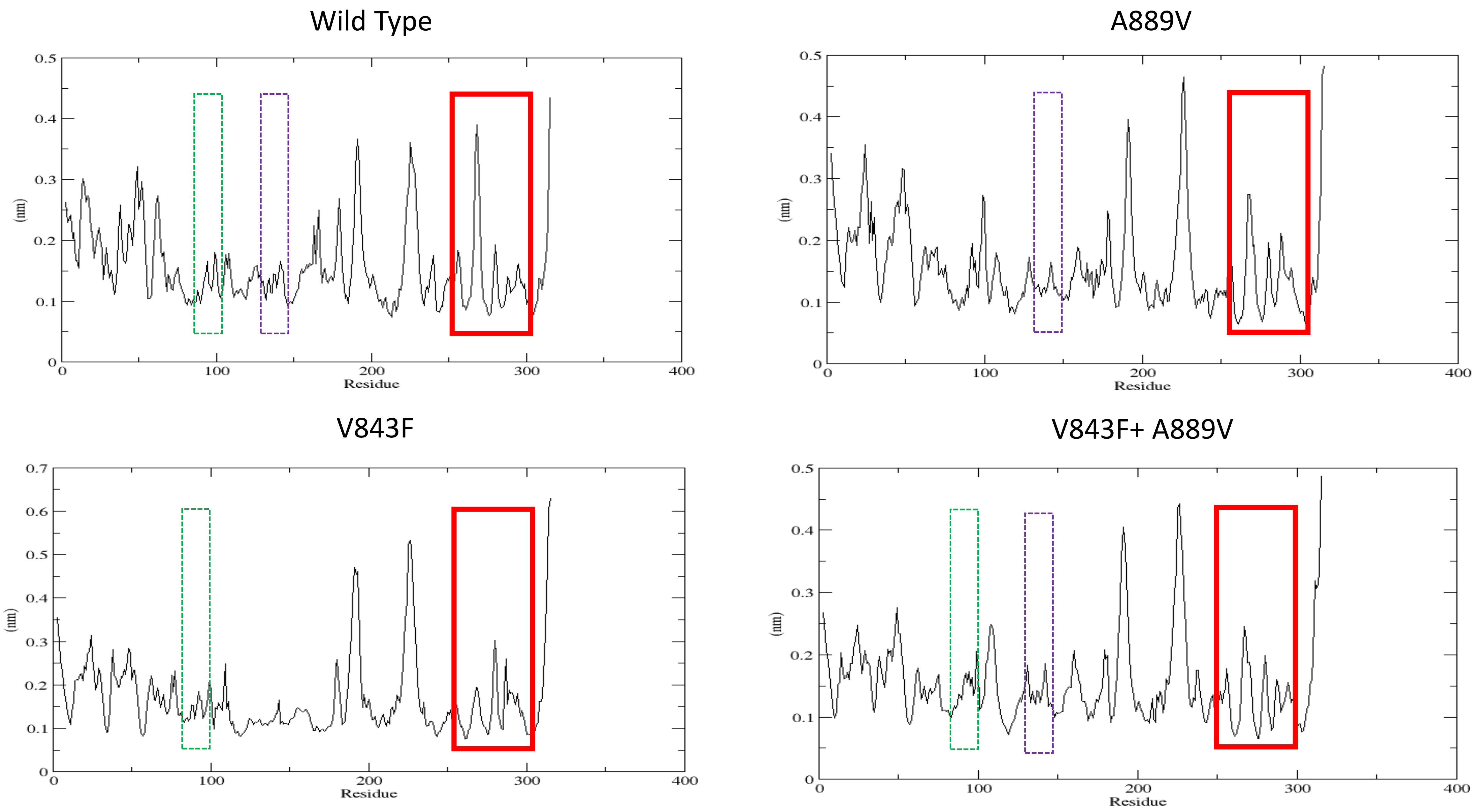

Root-Mean-Square Fluctuations (RMSF) of the GRL0617 docked with wild type and Mutant PLPros. Green and Violet Boxes depict mutations in 96th/142th positions or V843F/ A889V mutations respectively

Radius of gyration (total and around axes)

Wild Type

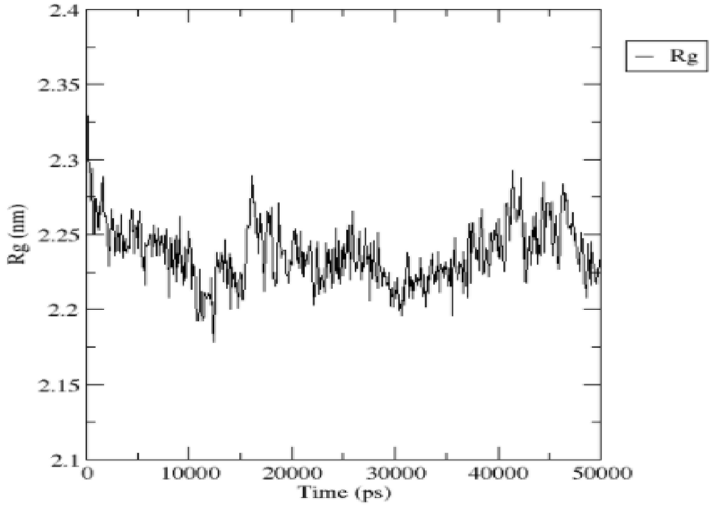

A889V

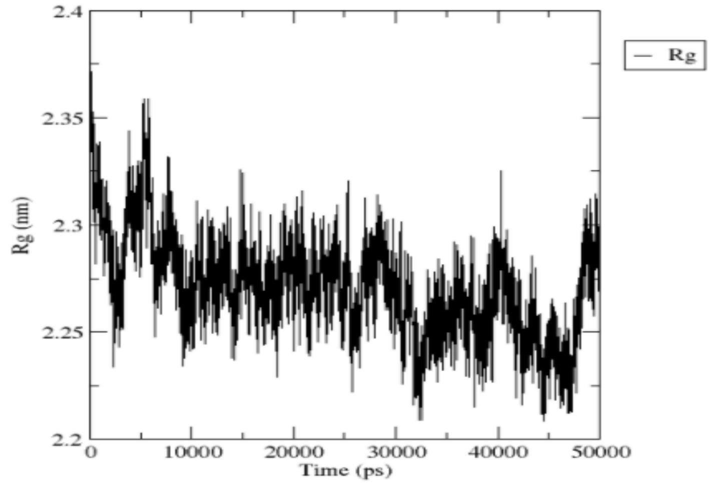

V843F

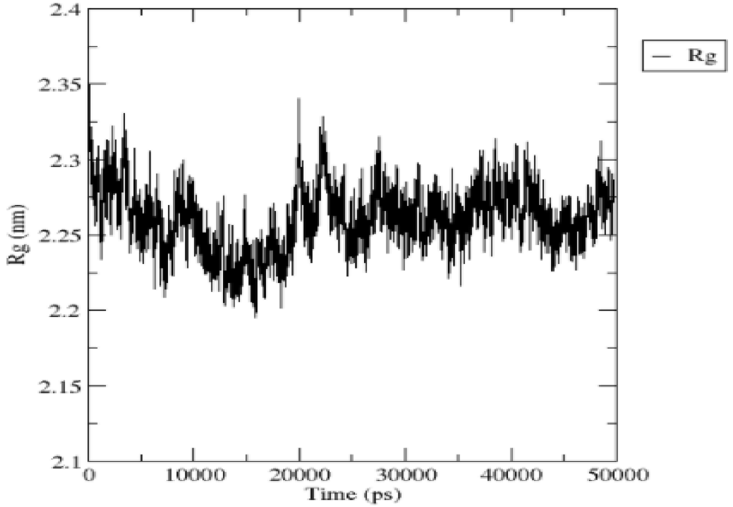

V843F+ A889V

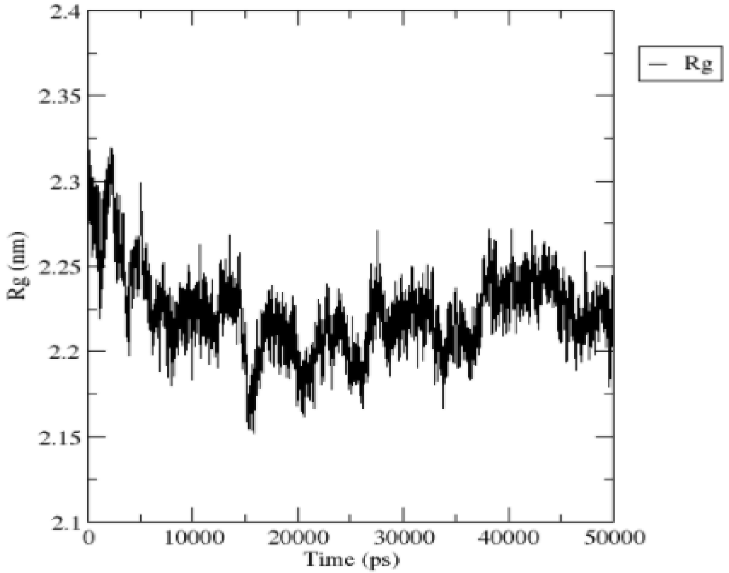

Supplement: Supplementary file 5 — Additional file 5: Supplementary File 5. Results from Molecular Dynamics (MD) Simulations. RMSD, RMSF and Rg values of GRL0617 bound wild type and mutant PLPro enzymes. [file 43141_2021_152_MOESM5_ESM.pdf]
